# Co-existence of antibiotic resistance and virulence factors in carbapenem resistant *Klebsiella pneumoniae* clinical isolates from Alexandria, Egypt

**DOI:** 10.1186/s12866-024-03600-1

**Published:** 2024-11-11

**Authors:** Aya T. El-kholy, Mohammed A. El-Kholy, Hoda Omar, Elsayed Aboulmagd

**Affiliations:** 1 College of Pharmacy, Arab Academy for Science, Technology and Maritime Transport, Alamein, Egypt; 2 Department of Microbiology and Biotechnology, Clinical and Biology Sciences Division, College of Pharmacy, Arab Academy for Science, Technology and Maritime Transport (AASTMT), Abu Qir Campus, P.O. Box 1029, Alexandria, Egypt; 3 Department of Microbiology and Immunology, Faculty of Pharmacy, Alexandria University, Alexandria, Egypt

**Keywords:** Carbapenem-resistant *Klebsiella pneumoniae*, Co-existence, Carbapenemases, Virulence factors

## Abstract

**Background:**

The emergence and spread of carbapenem resistance among *Enterobacteriaceae*, particularly *Klebsiella pneumoniae*, constitute a serious threat to public health, since carbapenems are the last line of defense in the treatment of life-threatening infections caused by drug-resistant *Enterobacteriaceae*. The current study investigated the co-existence of different virulence factors and carbapenemases in carbapenem-resistant *Klebsiella pneumoniae* clinical isolates from Alexandria, Egypt.

**Results:**

Phenotypic characterization of virulence factors indicated that 41.5% of the isolates were strong biofilm producers, while hypermucoviscosity was detected in 14.9% of the isolates. All isolates harbored five or more virulence factor encoding genes. *entB*,* ycfM*, *mrkD* and *fimH* were detected in all isolates, while only one isolate was negative for *ybtS*. *uge*, *iutA*, *rmpA* and *kpn* were detected in 61 (64.8%), 55 (58.5%), 41 (43.6%) and 27 (28.7%) isolates, respectively, while all isolates lacked *magA* and *k2A*. Phenotypic detection of carbapenemases was explored by performing CarbaNP and mCIM/eCIM. CarbaNP test showed positive results in 98.9% of the isolates and positive mCIM tests were observed in all isolates, while 68 (72.3%) isolates showed positive eCIM tests. *bla*_NDM_ was the most prevalent carbapenemase encoding gene (92.5%) followed by the *bla*_OXA−48_ (51.1%), while *bla*_KPC_ was detected in only one (1.06%) isolate. *bla*_VIM_, *bla*_IMP_ and *bla*_GES_ were not detected in any of the tested isolates.

**Conclusions:**

The widespread of carbapenem-resistant *Klebsiella pneumoniae* represents a major problem in health care settings. A significant association between certain virulence factors and carbapenemase-encoding genes was observed. Antibiotic stewardship programs and infection control policies should be effectively implemented especially in hospitals to limit the spread of such highly virulent pathogens.

**Supplementary Information:**

The online version contains supplementary material available at 10.1186/s12866-024-03600-1.

## Background

*Klebsiella pneumoniae* (*K. pneumoniae*) is a Gram-negative, encapsulated, non-motile bacterium found in the environment. It is a real source of nosocomial infections, which can result in a variety of infections, such as urinary tract infections, pneumonia, septicemia, and meningitis. Infections caused by *K. pneumoniae* are becoming challenging to treat, especially blood stream infections [1]. Worldwide, hospital-acquired infections were reported to be 8.7%, with *K. pneumoniae* accounting for almost 10% of cases [2]. Human oropharynx and gastrointestinal tract mucosal surfaces are colonized by *K. pneumoniae.* Once the bacterium enters the body, it may exhibit significant levels of virulence and antibiotic resistance [3, 4]. It shows resistant to the main antibiotic classes through production of a variety of β-lactamases, over-expression of efflux-pump systems, altering antibiotic targets and mutation in outer membrane permeability. It can easily acquire and disseminate genetic resistance determinants [5].

Virulence of *K. pneumoniae* is provided by a variety of factors that can lead to severity of infections and development of antibiotic resistance. The most crucial component of its pathogenicity is the polysaccharide capsule, which protects the bacterium from the host’s opsonophagocytosis and serum killing processes. Lipopolysaccharides that cover the outer surface are the second virulence factor and they cause an inflammatory cascade in the host, which has been linked to the sequelae of sepsis and septic shock. In addition, fimbriae allow the pathogen to adhere to host target cells [6, 7].

*K. pneumoniae* can be divided into two types: classic *K. pneumoniae* (cKp) and hypervirulent *K. pneumoniae* (hvKp). HvKp, an emerging pathogen, has higher pathogenicity than cKp. This type is unique in that it is obtained in the community and can cause severe invasive infections with metastatic features [8]. HvKP isolates exhibit a unique hypermucoviscosity phenotype when cultivated on agar plates, as confirmed by string test [9].

Siderophores are iron-acquisition molecules that are also considered as virulence factors, allowing the pathogenic microorganism to spread. *K. pneumoniae* has four distinct siderophores: aerobactin, enterobactin, salmochelin, and yersiniabactin. Enterobactin has the greatest affinity for iron and is seen in either classical or hypervirulent strains, making it the major iron absorption system [10, 11]. In addition, *K. pneumoniae* can produce biofilms inside catheters and other indwelling devices. Biofilms may contribute to colonization of the gastrointestinal, respiratory and urinary tracts and the development of invasive infections especially in immunocompromised patients [12].

Another virulence factor of *K. pneumoniae* that allows the bacteria to survive in environments with limited nutrients is urease. Urease is an essential enzyme capable of hydrolyzing urea into ammonia and carbon dioxide, which provides growth with nitrogen [13]. Urea hydrolysis raises the local pH and precipitates inorganic ions that are insoluble at high pH. This kind of precipitation can encourage the production of biofilms and cause encrustation on urinary catheters [12].

After extended-spectrum β-lactamase-producing *K. pneumoniae* strains, carbapenem-resistant *K. pneumoniae* (CRKP) isolates became a serious global public health concern, leading to high morbidity and mortality rates [14]. The worldwide health care system is currently burdened by a high incidence of carbapenem-resistant *Enterobacteriaceae* (CRE), particularly *K. pneumoniae* and *Escherichia coli* isolates [15]. CRKP has recently spread globally, including Egypt with prevalence rates between 48.1 and 100% [16, 17].

Carbapenemases are β-lactamase enzymes that can hydrolyze carbapenems and they are classified into three types using the Ambler classification system. Classes A and D are serine β-lactamases such as *K. pneumoniae* carbapenemase (KPC) and oxacillinase-48 (OXA-48), while class B are metallo-β-lactamases such as imipenemase metallo-β-lactamase (IMP), Verona integron-encoded metallo-β-lactamase (VIM) and New Delhi metallo-β-lactamase (NDM) [18, 19]. Prevalence rates of KPC, NDM and OXA-48 genes among *K. pneumoniae* isolates in Egypt vary between 0–95.8%, 20.9–100%, and 0–80.65%, respectively [17].

Polymyxins (colistin and polymyxin B) are the most commonly utilized antimicrobials in the fight against CRKP [20]. Detection of colistin resistance among *K. pneumoniae* isolates is considered a global threat, particularly because of the limited antimicrobial options available and the high mortality rate associated with these infections [21].

The current study investigated the co-existence of certain virulence factors and different carbapenemases in CRKP clinical isolates collected from different healthcare settings in Alexandria, Egypt.

## Materials and methods

### Bacterial isolates

A total of 94 non-duplicate CRKP clinical isolates were collected from Mabaret El Asafra Laboratories, Alexandria, from January 2021 to September 2021. They were obtained from various clinical specimens from Egyptian patients: blood (*n* = 51), wound swab (*n* = 13), tracheal aspirate (*n* = 8), sputum (*n* = 8), urine (*n* = 8), bronchoalveolar lavage (*n* = 5) and catheter (*n* = 1). The isolates were identified up to the species level by VITEK^®^ 2 automated compact system.

### Antimicrobial susceptibility testing (AST)

The sensitivity of the isolates towards different antimicrobial agents was determined by the following methods:

1. **VITEK 2 compact system** N222 card (bioMérieux, Marcy l’Etoile, France) was used to determine the sensitivity of the isolates to the following seven antimicrobial agents: aztreonam, ceftazidime, piperacillin, piperacillin-tazobactam, ticarcillin, ticarcillin-clavulanic acid and trimethoprim-sulfamethoxazole.
2. **Kirby-Bauer test** The susceptibility of the isolates to 13 antimicrobial agents was determined by agar disc diffusion on Müller-Hinton agar (MHA) (Oxoid Ltd., Basingstoke, UK) according to Clinical and Laboratory Standards Institute (CLSI 2021 M100) recommendations. Antibiotics (Oxoid Ltd., Basingstoke, UK) used were as follows: ampicillin, amoxicillin-clavulanic acid, cefepime, imipenem, meropenem, ertapenem, doripenem, gentamicin, tobramycin, amikacin, ciprofloxacin, levofloxacin, and doxycycline. Isolates were classified as susceptible, moderately resistant, and resistant according to the CLSI breakpoints [22].
3. **Colistin broth disk elution** The susceptibility to colistin was determined by broth disc elution method according to CLSI guidelines [22]. Briefly, three to five colonies were suspended in sterile saline 0.9% to adjust the turbidity at 0.5 McFarland. For each isolate, four tubes containing 10 mL cation-adjusted Müller-Hinton broth (Oxoid Ltd., Basingstoke, UK) were labelled 0 (as control), 1, 2 and 4 µg/mL. One, two or four colistin (10 µg) discs were transferred to the tubes labeled 1 µg/mL, 2 µg/mL, or 4 µg/mL, respectively. No discs were added to the control tube (0 µg/mL). The tubes were inoculated with the tested isolates to get a final inoculum of approximately 7.5 × 10^5^ CFU/mL. The tubes were incubated at 35 °C for 16–20 h. MIC value was read as the lowest concentration of colistin that completely inhibits the growth of the tested isolate and interpreted according to the EUCAST guidelines where the tested isolates were categorized into susceptible (MIC < 2 µg/mL) and resistant (MIC ≥ 4 µg/mL) [23].
4. **Determination of the minimum inhibitory concentration (MIC)** MIC values of ertapenem, imipenem, meropenem and colistin were determined using broth microdilution method [24]. The tested antimicrobial agents were two fold serially diluted in a 96-well microtiter plate (BD Falcon; Fisher Scientific, USA). The final inoculum of each isolate was approximately 5 × 10^5^ CFU/mL. The plates were incubated at 35 °C for 18–20 h. MIC values were interpreted according to the CLSI breakpoints [22].

### Phenotypic detection of virulence factors

1. **Biofilm production assay** Overnight cultures of the tested isolates in sterile tryptic soya broth (TSB) were diluted 1:100 in the same medium supplemented with 1% glucose. Two hundred µL of each bacterial suspension were transferred into sterile flat-bottomed 96-well polystyrene microtiter plates (Citotest, China) then incubated for 24 h at 37 °C without agitation. After incubation, the wells were washed three times with phosphate buffer saline (PBS), fixed with 99% methanol, stained with 150 µl of 2% crystal violet solution for 15 min, and finally re-solubilized with 150 µl of 33% glacial acetic acid. The absorbance of the re-solubilized solution was measured at 620 nm using the ELISA plate reader (Tecan Infinite F50 Microplate Reader, Switzerland). An uninoculated medium was used as a negative control in each plate. The clinical isolates as well as controls were tested in triplicate. Results were interpreted according to Stepanovic et al. [25].
2. **Hypermucoviscosity testing** Bacterial isolates were assessed for hypermucoviscosity phenotype using a string test [11]. A positive string test [hypermucoviscous (HMV) isolate] is defined as the formation of viscous string of > 5 mm in length when a loop is used to stretch the colony grown overnight on blood agar plate at 37 °C.
3. **Phospholipase C production (lecithinase production)** Phospholipase C production assay was carried out by cultivating the isolates on egg yolk agar. Positive results were considered depending on the formation of a clear zone around the colony [26].

### Phenotypic detection of carbapenemases

1. **CarbaNP test** CarbaNP test was performed according to the CLSI guidelines [22] to detect the production of different carbapenemases. One µl of an overnight cultured isolate was suspended in two Eppendorf tubes (A and B) containing 100 µl of bacterial protein extraction reagent (Thermo Scientific Pierce, Rockford, IL, USA), 100 µl of CarbaNP revealing solution and 6 mg/mL imipenem in only tube B. After incubation for 2 h at 37 °C, both tubes A and B were visually inspected for color change. The carbapenemase activity was detected by a color change from red to light orange, dark yellow, or yellow in tube B, resulting from the hydrolysis of imipenem into a carboxylic derivative, and leading to a decrease of the pH value.
2. **Modified carbapenem inactivation method (mCIM) and EDTA-modified carbapenem inactivation method (eCIM)** mCIM and eCIM methods were performed according to the CLSI guidelines [22]. Briefly, one µl of each isolate was suspended in a 2-mL TSB. Another 1 µl of each isolate was suspended in 2 mL TSB supplemented with EDTA at a final concentration of 5 mM. Meropenem disc (10 µg) was transferred into each tube, and the tubes were incubated at 35^◦^C for 4 h. After incubation, the discs were removed and placed onto MHA plates that were recently inoculated with 0.5 McFarland suspension of a carbapenem-susceptible *E. coli* ATCC^®^ 29522 indicator strain and incubated at 37 °C for 24 h. For mCIM, a clear zone diameter of 6–15 mm was considered as carbapenemase producer isolate. A ≥ 5 mm increase in zone diameter for eCIM versus the zone diameter for mCIM was considered as metallo-β-lactamase producer isolate.

### DNA extraction and multiplex PCR

1. **DNA extraction** DNA extract was prepared by suspending 7–9 colonies of each isolate in 200 µl of sterile deionized water. The bacterial suspensions were heated at 98 °C for 10 min, followed by centrifuging the cell extract for 5 min at 15,000×g at 4 °C using a cooling centrifuge (Finsen, Bunsen, Spain). The supernatant was removed and preserved at -20 °C to be used as the template DNA for PCR [27].
  For multiplex PCR, MyTaq™ HS Red Mix (Bioline Reagents Ltd United Kingdom) kit was used according to the manufacturer instructions. Thermal cycling conditions of the multiplex PCR were as follows: single cycle as initial denaturation (2 min at 95 °C), followed by 30 cycles: denaturation (30 s at 95 °C), annealing (15 s at 58 °C), and extension (30 s at 72 °C) [28].
2. **Genotypic detection of virulence determinants** Eleven virulence factor encoding genes were investigated by four different multiplex PCRs using previously published primers [29–34]. Reaction 1 includes the detection of *kpn* (like fimbrial adhesion), *entB* (enterobactin siderophore system), *ycfM* (outer membrane lipoprotein) and *k2A* (specific to K2 capsule serotype). Reaction 2 includes the detection of *uge* (uridine diphosphate galacturonate 4-epimerase), *iutA* (aerobactin siderophore system) and *magA* (mucoviscosity-associated gene A). Reaction 3 includes the detection of *rmpA* (the regulator of mucoid phenotype A) and *ybtS* (yersiniabactin siderophore system). Reaction 4 includes the detection of *mrkD* (the type 3 fimbrial adhesion) and *fimH* (type 1 fimbriae). The primers used in the current study are listed in (Supplemental Table S1).
3. **Genotypic detection of carbapenemases encoding genes** Six carbapenemases (*bla*_VIM_, *bla*_KPC_, *bla*_NDM_, *bla*_OXA−48_, *bla*_IMP_ and *bla*_GES_) were investigated by two multiplex PCRs using previously published primers [35, 36]. The first reaction was used to detect *bla*_VIM_, *bla*_NDM_ and *bla*_OXA−48_, while, the second reaction was used to explore *bla*_KPC_, *bla*_IMP_ and *bla*_GES_. PCR products were analyzed by agarose gel electrophoresis (2%) in the presence of 50 bps DNA ladder (GeneDirex, Taiwan). The primers used in the current study are listed in (Supplemental Table S1).

### Statistical analysis of the data

Data were analyzed using the Shapiro-Wilk test, Chi-square test and Fisher’s Exact using IBM SPSS software package version 20.0. **(**Armonk, NY: IBM Corp**).** Spearman coefficient was used to correlate between two distributed abnormally quantitative variables. The significance of the obtained results was judged at the 5% level.

## Results

In the current study, 94 *K. pneumoniae* clinical isolates were collected from various clinical specimens: blood (*n* = 51), wound swab (*n* = 13), tracheal aspirate (*n* = 8), sputum (*n* = 8), urine (*n* = 8), bronchoalveolar lavage (*n* = 5) and catheter (*n* = 1).

### Antimicrobial susceptibility testing

**Fig. 1:**
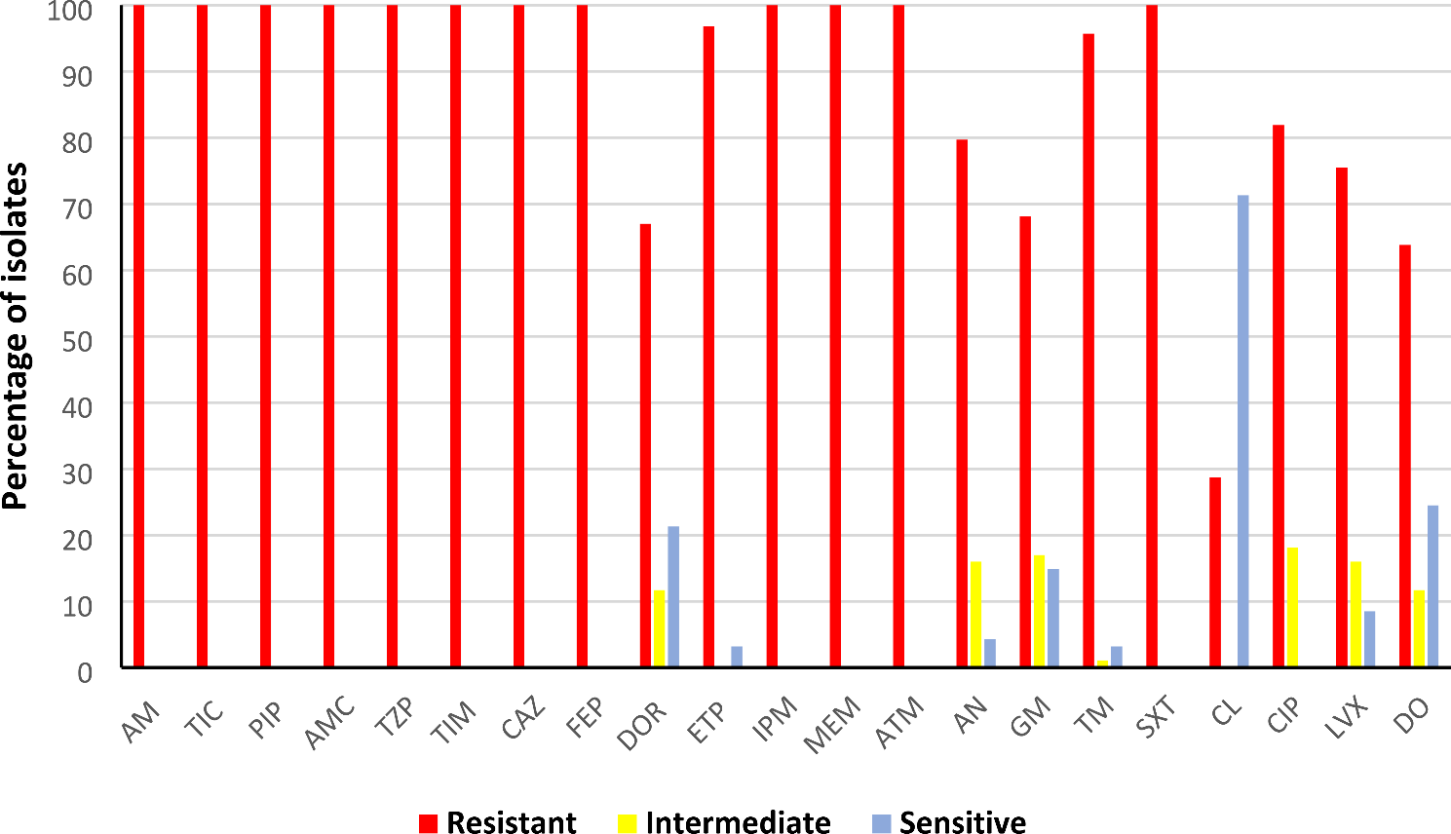
Antimicrobial susceptibility profiles of the CRKP clinical isolates. AM, ampicillin; TIC, ticarcillin; PIP, piperacillin; AMC, amoxicillin-clavulanate; TZP, piperacillin-tazobactam; TIM, ticarcillin-clavulanate; CAZ, ceftazidime; FEP, cefepime; DOR, doripenem; ETP, ertapenem; IPM, imipenem; MEM, meropenem; ATM, aztreonam; AN, amikacin; GM, gentamicin; TM, tobramycin; SXT, trimethoprim/sulfamethoxazole; CL, colistin; CIP, ciprofloxacin; LVX, levofloxacin; DO, doxycycline

Ninety-four CRKP isolates showed 100% resistance to penicillins, β-lactam/β-lactamase inhibitor combinations, cephalosporins, and aztreonam, in addition to imipenem and meropenem, while the susceptibility rates of doripenem and ertapenem were 21.2% and 3.1%, respectively. The aminoglycosides (amikacin, gentamicin and tobramycin), fluoroquinolones (ciprofloxacin and levofloxacin), trimethoprim-sulfamethoxazole and doxycycline showed resistance rates ranging from 63.8 to 95.7%. On the other hand, colistin revealed the highest activity against the isolates, where 72.2% of isolates were susceptible as shown in (Fig. 1) and (Supplemental file S2).

The MIC ranges, MIC_50_ and MIC_90_ values of various antimicrobial agents against the isolates are depicted in Table 1. The MIC_50_ and MIC_90_ values for imipenem and meropenem were ≥ 64 µg/mL. Regarding ertapenem and colistin, MIC_50_ values were > 64 µg/mL and 2 µg/mL, while MIC_90_ values were > 64 µg/mL and 64 µg/mL, respectively (Table 1).

**Table 1:** MIC ranges, MIC_50_, and MIC_90_ of tested carbapenems and colistin against *K. pneumoniae* isolates

Antimicrobial agents	MIC ranges	MIC_50_	MIC_90_
Imipenem	16 - > 64	> 64	> 64
Meropenem	16 - > 64	> 64	> 64
Ertapenem	< 0.25 - > 64	> 64	> 64
Colistin	0.5 - > 64	2	64

### Phenotypic detection of virulence factors

Phenotypic characterization of CRKP virulence factors indicated that 14.9% of the isolates were HMV while 85.1% were classified as non-HMV (NHMV). On the other hand, 41.5% were classified as strong biofilm producers, while 39.4% showed moderate production and 19.1% were considered as weak biofilm producers as shown in (Supplemental file S3).

**Fig. 2:**
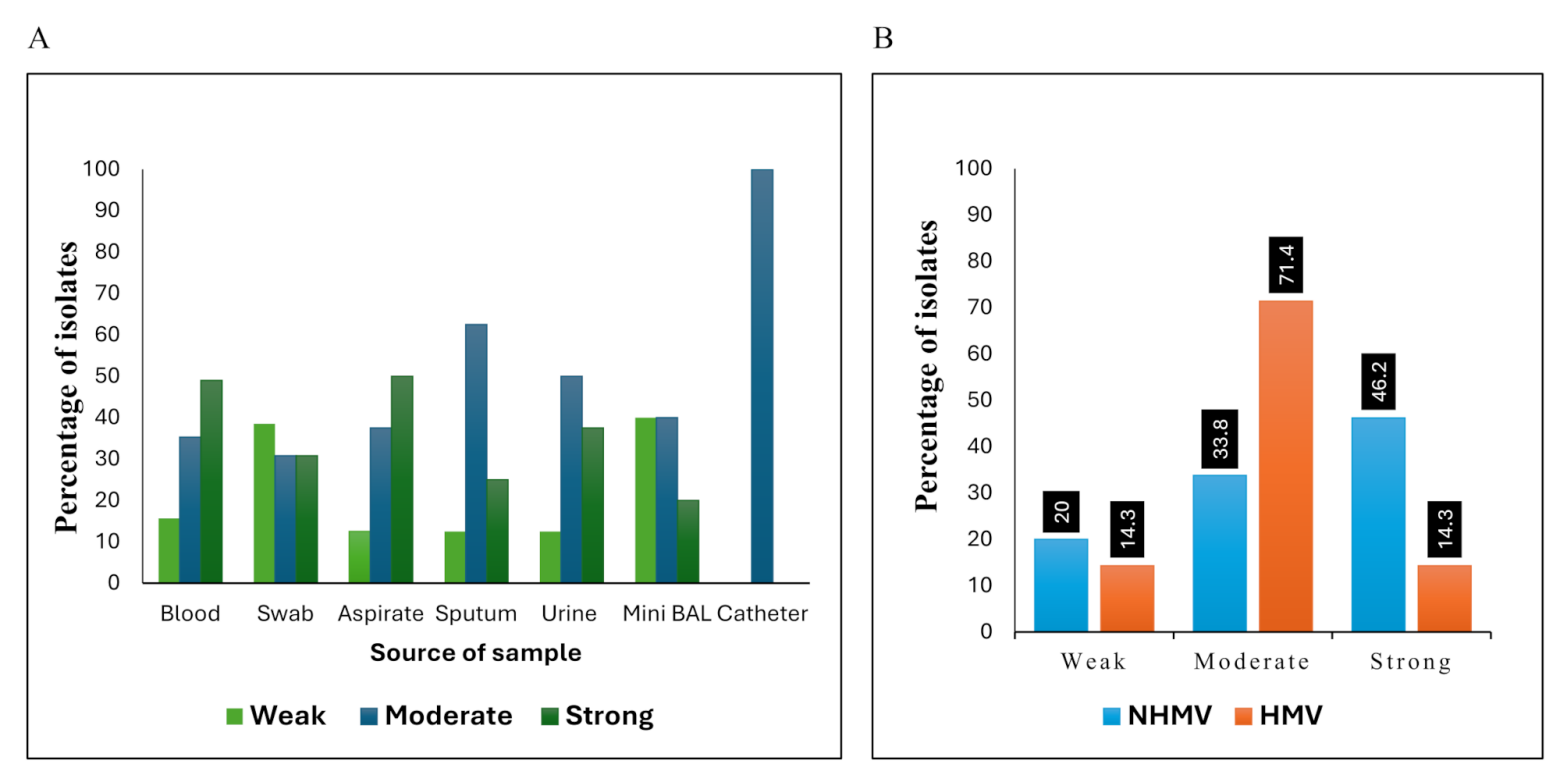
Relation of biofilm production capacity with sample source (**A**) and HMV phenotype (**B**)

The level of biofilm formation was not significantly related to the source of the sample (*p*-value = 0.649). Strong biofilm producers were detected in 50% of isolates from aspirate specimens, 49% of blood, 37.5% of urine, 30.8% of swabs, 25% of sputum and 20% of bronchoalveolar lavage. There is a statistically significant difference (*p*-value = 0.024) between the capacity of the isolates to produce biofilm and their HMV determined by string test (Fig. 2). In addition, all tested isolates did not exhibit phospholipase C (lecithinase) activity.

### Genotypic detection of virulence determinants

In the current study, all isolates harbored *entB*,* ycfM*, *mrkD* and *fimH* as shown in Table 2, while *magA* and *k2A* were not detected in any isolate, indicating that the tested isolates were considered as non-K1/K2 strains. Only one isolate was negative for *ybtS*. The *uge*, *iutA*, *rmpA* and *kpn* genes were detected in 61, 55, 41 and 27 isolates, respectively. It was found that biofilm formation was more noticeable among *rmpA*-harboring isolates (*p*-value = 0.004).

**Table 2:** Detection of carbapenemase and virulence factor encoding genes in CRKP clinical isolates

No. of isolates	Code of isolates	Virulence factor encoding genes									Carbapenemase encoding genes	
		*k* *pn*	*entB*	*ycfM*	*uge*	*iutA*	*ybtS*	*rmpA*	*mrkD*	*fimH*		
18	KP17, KP18, KP21, KP23, KP25, KP30, KP31, KP33, KP36, KP38, KP39, KP46, KP53, KP58, KP63, KP66, KP83, KP84	-	+	+	+	-	+	-	+	+	*bla*_NDM_	
12	KP2, KP4, KP11, KP13, KP14, KP47, KP49, KP54, KP62, KP70, KP85, KP86	-	+	+	+	+	+	+	+	+	*bla*_NDM_ + *bla*_OXA−48_	
8	KP12, KP29, KP40, KP42, KP64, KP71, KP72, KP79	-	+	+	+	+	+	+	+	+	*bla*_NDM_	
7	KP1, KP10, KP16, KP51, KP55, KP59, KP74	+	+	+	-	+	+	+	+	+	*bla*_NDM_ + *bla*_OXA−48_	
5	KP35, KP45, KP67, KP88, KP92	-	+	+	-	+	+	+	+	+	*bla*_NDM_ + *bla*_OXA−48_	
5	KP76, KP78, KP82, KP90, KP94	-	+	+	-	-	+	-	+	+	*bla*_NDM_	
5	KP15, KP32, KP26, KP28, KP68	+	+	+	+	+	+	+	+	+	*bla*_NDM_ + *bla*_OXA−48_	
3	KP7, KP8, KP19	+	+	+	-	-	+	-	+	+	*bla*_NDM_ + *bla*_OXA−48_	
3	KP87, KP91, KP93	-	+	+	-	-	+	-	+	+	*bla*_NDM_ + *bla*_OXA−48_	
3	KP22, KP24, KP80	-	+	+	-	+	+	-	+	+	*bla*_NDM_	
3	KP20, KP27, KP37	+	+	+	+	+	+	-	+	+	*bla*_NDM_ + *bla*_OXA−48_	
2	KP57, KP75	-	+	+	-	+	+	+	+	+	*bla*_NDM_	
2	KP43, KP65	-	+	+	+	+	+	-	+	+	*bla*_NDM_	
2	KP60, KP73	-	+	+	+	+	+	-	+	+	*bla*_NDM_ + *bla*_OXA−48_	
2	KP34, KP6	+	+	+	+	-	+	-	+	+	*bla*_NDM_ + *bla*_OXA−48_	
2	KP44, KP61	+	+	+	+	-	+	-	+	+	*bla*_NDM_	
2	KP50, KP89	-	+	+	+	-	+	-	+	+	*bla*_NDM_ + *bla*_OXA−48_	
1	KP48	-	+	+	+	+	+	+	+	+	*bla*_OXA−48_	
1	KP77	+	+	+	-	-	+	-	+	+	*bla*_OXA−48_	
1	KP69	-	+	+	+	+	+	-	+	+	*bla*_OXA−48_	
1	KP56	+	+	+	-	-	+	-	+	+	*bla*_NDM_	
1	KP3	-	+	+	-	+	+	-	+	+	*bla*_NDM_ + *bla*_OXA−48_	
1	KP5	+	+	+	-	+	+	-	+	+	*bla*_NDM_ + *bla*_OXA−48_	
1	KP9	-	+	+	+	+	-	+	+	+	*bla*_NDM_	
1	KP52	+	+	+	+	-	+	-	+	+	*bla*_KPC_	
1	KP41	+	+	+	+	+	+	-	+	+	*bla*_NDM_	
1	KP81	-	+	+	+	-	+	-	+	+	None	

### Phenotypic detection of carbapenemase encoding genes

Ninety-three isolates (98.9%) showed positive CarbaNP test (Table 3). Ten isolates (10.75%) developed the positive result in < 15 min, while 83 isolates turned positive after 2 h of incubation. On the other hand, only one isolate developed a non-interpretable result. In addition, all isolates exhibited carbapenemase activity that was observed using mCIM test, while, metallo-β-lactamases were detected in 72.3% of the isolates depending on eCIM results (Table 3) and as shown in (Supplemental file S3).

**Table 3:** Prevalence of different carbapenemase encoding genes in CRKP clinical isolates

Resistance genes (number of isolates)	Phenotypic characterization		
	CarbaNP	mCIM	eCIM
*bla*_NDM_ (44 )	44 (100%)	44 (100%)	43 (97.7%)
*bla*_OXA−48_ (5)	5 (100%)	5 (100%)	3 (60%)
*bla*_KPC_ (1)	1 (100%)	1 (100%)	0 (0%)
*bla*_NDM_ + *bla*_OXA−48_ (43)	43 (100%)	43 (100%)	21 (48.8%)

### Genotypic detection of carbapenemase encoding genes

Out of the six carbapenemase-encoding genes investigated, *bla*_NDM_ and *bla*_OXA−48_ were detected in 87 (92.5%) and 48 (51.1%) isolates, respectively (Table 2). Furthermore, co-existence of *bla*_NDM_ and *bla*_OXA−48_ were detected in 43 (45.7%) isolates. The co-existence is significantly associated with doripenem-resistant isolates (*p*-value = 0.003). Only one isolate harbored *bla*_KPC_, while *bla*_IMP_, *bla*_GES_ and *bla*_VIM_ were not detected in any of the isolates (Table 2).

### Association between the virulence factors and antibiotic resistance

The current study showed diversity in the virulence profiles among the isolates and co-existence between different virulence factors and antibiotic resistance was investigated. The co-existence of *bla*_NDM_ and *bla*_OXA−48_ was significantly associated with the number of virulence factor encoding genes detected in each isolate (*p-*value < 0.001). It was observed that *bla*_OXA−48_ encoding gene was highly correlated with different virulence factor encoding genes including *kpn*, uge, *iutA* and *rmpA*. On the contrary, *bla*_NDM_ was not significantly related to any of the detected virulence factor encoding genes (Table 4).

**Table 4:** Association between *K. pneumoniae* virulence factor and carbapenemase encoding genes

	Number of isolates (%)			
	***bla***_**OXA−48**_		***bla***_**NDM**_	
	**Positive**	**Negative**	**Positive**	**Negative**
***kpn***				
Positive	21 (77.8%)	6 (22.2%)	25 (92.6%)	2 (7.4%)
Negative	27 (40.3%)	40 (59.7%)	62 (92.5%)	5 (7.5%)
*p*-value	0.001*		1.000	
***uge***				
Positive	26 (42.6%)	35 (57.4%)	57 (93.4%)	4 (6.6%)
Negative	22 (66.7%)	11 (33.3%)	30 (90.9%)	3 (9.1%)
*p*-value	0.026*		0.693	
***iutA***				
Positive	37 (67.3%)	18 (32.7%)	53 (96.4%)	2 (3.6%)
Negative	11 (28.2%)	28 (71.8%)	34 (87.2%)	5 (12.8%)
*p*-value	< 0.001*		0.122	
***rmpA***				
Positive	29 (70.7%)	12 (29.3%)	40 (97.6%)	1 (2.4%)
Negative	19 (35.8%)	34 (64.2%)	47 (88.7%)	6 (11.3%)
*p*-value	0.001*		0.132	
***ybtS***				
Positive	48 (51.6%)	45 (48.4%)	86 (92.5%)	7 (7.5%)
Negative	0 (0%)	1 (100%)	1 (100%)	0 (0%)
*p*-value	0.489		1.000	

*The *p*-values indicate significance where *p* < 0.05

A significant association between colistin resistance and the ability of the isolates to produce biofilm was observed (*p*-value = 0.044). It was shown that colistin-resistant *K. pneumoniae* isolates were moderate or weak biofilm producers, while most colistin-susceptible *K. pneumoniae* isolates (49.3%) were strong biofilm producers. On the other hand, a significant association between doripenem resistance and strong ability to produce biofilm was detected (*p*-value = 0.010).

## Discussion

*K. pneumoniae* causes one-third of Gram-negative infections and is linked with carbapenem resistance worldwide [37]. Hence, the importance of the current study is to investigate the co-existence of certain virulence factors and different carbapenemases in CRKP clinical isolates collected from different healthcare settings in Alexandria, Egypt.

Carbapenem resistance is increasing in *K. pneumoniae* isolates collected from Blood stream infections. In the current study, 94 CRKP isolates were collected from different clinical specimens. Most of the isolates were from blood specimens (51 isolates), while only one isolate was isolated from catheter. Recent studies from Egypt [38, 39] and Italy [40] have shown similar results, where most of the isolates were obtained from blood specimens.

The antimicrobial susceptibility profile revealed that all tested isolates were non-susceptible to imipenem, meropenem, and ertapenem, while 21.2% were susceptible to doripenem. Doripenem is a recently introduced 1-b-methyl-carbapenem with a specific side chain substitution that enhances its activity [41]. Due to its modified structure, doripenem is presumed to have improved cell penetration, particularly in Gram-negative bacteria, owing to its ability to interact more effectively with porin channels, thereby facilitating better traversal of the outer membrane compared to other carbapenems. Furthermore, doripenem exhibits reduced interactions with carbapenemases, thereby mitigating or reducing the likelihood of resistance development associated with these enzymes. This reduced cross-resistance with other carbapenems makes doripenem a valuable therapeutic alternative when other carbapenems may be ineffective against Gram-negative bacilli (GNB) clinical isolates [42]. Additionally, doripenem is neither available nor commonly prescribed in Egypt, which could explain the observed better sensitivity profile in the current study.

Colistin is considered as the last resort therapeutic option for the treatment of severe CRKP infections, however, increasing microbial resistance and related serious side effects are worrisome [43]. In the current study, the frequency of colistin-resistant isolates was relatively high (28.7%.). Comparable prevalence rates were previously reported in Egypt [44], Saudi Arabia [45] and Thailand [46] where the resistance rates exceeded 20% among CRKP isolates. Such colistin resistance rates may be attributed to the overuse of colistin and the lack of commitment to the implementation of an effective antimicrobial stewardship program in Egypt. Nevertheless, lower prevalence rates of colistin-resistance (8.8–14%) were reported in other previous studies conducted in Egypt [38, 39, 47] and worldwide [48, 49] .

The ability of microorganisms to produce biofilm is considered an important virulence trait, where it is estimated that 65–80% of bacterial infections are biofilm-related [50, 51]. Biofilm formation may result in an increase in resistance to different antimicrobial agents [52, 53]. In the present study, all investigated isolates were biofilm producers with different capacities. Similarly, Ragheb et al. [54] reported that all studied isolates were categorized as biofilm producers. Despite the lack of a significant association between antimicrobial resistance and biofilm formation, their co-existence is regarded as a problematic feature of the isolates analyzed.

HMV *K. pneumoniae* can cause severe infection in critically ill patients, including those in intensive care units [55]. In the present study, HMV isolates represented 14.9% of the isolates and this result is in agreement with previously published studies that showed prevalence rates of about 13.8% in Egypt [56] and 15.8% in Saudi Arabia [57]. On the contrary, other studies recorded variable prevalence rates ranging from 1% in China [58] to 40% in Egypt [59]. Furthermore, CRKP isolates with HMV phenotype were observed to be weak biofilm producers which may be attributed to the negative impact of exopolysaccharides synthesis on CRKP isolate fitness [60]. Moreover, this demonstrates that the presence of capsular polysaccharides decreases bacterial adherence, most likely by masking of the fimbrial adhesins [61, 62]. In addition, Di Domenico et al. [40] reported that the HMV phenotype showed a significant reduction in biofilm formation when compared to NHMV strains.

Among the explored virulence factor encoding genes, *entB*,* ycfM*, *mrkD* and *fimH* genes were detected in all isolates, while only one isolate was negative for *ybtS*. These results are in agreement with those reported by Naga in Egypt [63], Aljanaby and Alhasani in Iraq [64] and Zhan et al. in china [65]. Among the isolates, 64.8%, 58.5% and 43.6% harbored *uge*, *iutA* and *rmpA* respectively. Comparable prevalence rates were previously published in Egypt [63, 66, 67]. On the other hand, a significant association between biofilm formation and the presence of *rmpA* gene was recorded. Similarly, Zheng et al. [68] reported that biofilm formation was more obvious among *rmpA*-harboring isolates. In addition, *kpn* gene that encodes for fimbrial adhesion was found in 28.7% of the isolates. On the contrary, many previous studies reported higher prevalence rates ranging from 45.9 to 97.7% [28, 69–72]. Moreover, capsule encoding genes *magA* and *k2A* were not detected in any of the isolate, indicating that all the clinical isolates were considered as non-K1/K2 strains. This result is concordant with previous reports from Egypt [73], Iran [74] and Turkey [28].

Phenotypic detection of carbapenemases was explored by performing CarbaNP and mCIM/eCIM. CarbaNP test showed positive results in 98.9% of the isolates. Variable prevalence rates ranging from 40.5 to 90% were recently reported [75–77]. For detection and differentiation between the various types of carbapenemases, mCIM/eCIM assay was carried out. Positive mCIM tests were observed in all isolates, while 68 (72.3%) isolates showed positive eCIM tests. These result are aligned with the percentages mentioned by Shen et al. [78]. Surprisingly, eCIM failed to explore metallo-β-lactamase production in 22 isolates that co-harbor *bla*_NDM_ and *bla*_OXA−48_. This finding is in accordance with that previously reported in Egypt [47]. It was suggested that the production of a serine carbapenemase is likely disguised as the inhibitory impact of EDTA on the accompanying metallo-β-lactamase. Furthermore, such undetected metallo-β-lactamases activity may be due to the lack of gene expression or the genes were truncated, resulting in nonfunctional enzymes [79].

Molecular characterization of carbapenemases in the isolates demonstrated at least one gene in all the investigated isolates except KP 81. *bla*_NDM_ was the most prevalent gene (92.5%) followed by the *bla*_OXA−48_ (51.1%) and this prevalence was consistent with a previous study from Egypt [47], while El-Kholy et al. [80] and Zafer et al. [81] reported that *bla*_OXA−48_ as the most prevalent gene followed by *bla*_NDM_. It is well known that *bla*_NDM_ gene is carried on a number of easily mobile conjugative plasmids that are capable of horizontal gene transfer at inter- and intra-species levels, which possibly explains its predominance [82].

Co-existence of *bla*_*OXA*−48_ and *bla*_NDM_ genes was detected in 43 isolates (45.7%) and this co-existence extended resistance to different carbapenems where significant association with doripenem resistance was detected (*p*-value = 0.003). A low incidence rate of *bla*_KPC_ (1.06%) was identified in the current study, which is following Kamel et al. [83], Davoudabadi et al. [84] and Zafer et al. [81] who reported prevalence rates of 3.1%, 0% and 0%, respectively.

The association between antibiotic resistance and the presence of virulence factors plays an essential role in bacterial pathogenesis [29, 85–87]. The current study showed that there is a significant correlation between carbapenem resistance and virulence factor encoding genes (*p*-value ˂0.001). A significant association existed between doripenem-resistant isolates and strong abilities to produce biofilm (*p*-value = 0.010). Moreover, strong biofilm production was significantly associated (*p*-value = 0.044) with colistin sensitivity of the isolates (49.3%) compared to colistin resistance (22.2%). This result is following a previous study from Thailand [46]. Although biofilm production is thought to play a role in antibiotic resistance, this relationship may not always be directly proportional and could potentially be reversed. There are instances where isolates that produce strong biofilms appear to be more sensitive to antibiotics. One possible explanation for this is that bacteria shielded by biofilms may not rely on or require the same resistance mechanisms as planktonic cells [88]. The observed colistin sensitivity among strong biofilm producers in this study could be attributed to prior findings suggesting a possible loss of specific genomic regions involved in modifying bacterial lipopolysaccharides (LPS), which confer colistin resistance. These modifications have been linked to biofilm production in Enterobacteriaceae, providing a possible explanation for the inverse relationship between biofilm formation and colistin resistance [89, 90]. Further large-scale studies are required to elucidate the precise molecular mechanisms underlying this relationship between biofilm production and colistin sensitivity.

In conclusion, this study highlighted the association between different virulence factors and carbapenemase-encoding genes in CRKP clinical isolates. The alarmingly high prevalence of antibiotic resistance and high pathogenic capacity of CRKP necessitates the application of strict infection control measures as well as the implementation of an effective antimicrobial stewardship program. For a better understanding of coregulatory processes, active surveillance of antibiotic resistance and virulence determinant prevalence are strongly advocated. This will allow for a more educated approach to infection prevention and treatment regimens. Further studies are required to study the clonal relatedness disseminating in the health care facilities, as well as the impact of carbapenemases on fitness and virulence in *K. pneumoniae* clinical isolates.

## Electronic Supplementary Material

Below is the link to the electronic supplementary material.

Supplementary Material 1

Supplementary Material 2

Supplementary Material 3

## Data Availability

The datasets used and/or analyzed during the current study are available from the corresponding author on reasonable request.

## Abbreviations

CRKP: Carbapenem resistant *Klebsiella pneumoniae*
CRE: Carbapenem-resistant *Enterobacterales*
TSB: Tryptic soya broth
ATCC: American Type Culture Collection
CLSI: Clinical and Laboratory Standards Institute
EUCAST: European Committee on Antimicrobial Susceptibility Testing
TSB: Tryptic soy broth
OD: Optical density
PCR: Polymerase chain reaction
EDTA: Ethylenediaminetetraacetic acid
