# Supplementary material for: Co-existence of antibiotic resistance and virulence factors in carbapenem resistant Klebsiella pneumoniae clinical isolates from Alexandria, Egypt
Source: BMC Microbiol. 2024 Nov 11;24:466. doi: 10.1186/s12866-024-03600-1 (PMC11552214; doi:10.1186/s12866-024-03600-1)
Supplement: Supplementary file 1 — Supplementary Material 1 [file 12866_2024_3600_MOESM1_ESM.docx]

**Supplemental Table S1.** Primer sets used for the detection of carbapenemase and virulence factor encoding genes.

| **Carbapenemase encoding genes** | **Primer sequence (5'- 3')** | **Amplicon size (bps)** |
| --- | --- | --- |
| *bla*_VIM_ | F: GATGGTGTTTGGTCGCATATC  R: CGTCATGAAAGTGCGTGGAG | 202 |
| *bla*_NDM_ | F: GTTTGATCGTCAGGGATGGC  R: CTCATCACGATCATGCTGGC | 517 |
| *bla*_OXA-48_ | F: GGTAGCAAAGGAATGGCAAGAA  R: CGACCCACCAGCCAATCTTA | 611 |
| *bla*_KPC_ | F: CGCCAATTTGTTGCTGAAGG  R: CAGGTTCCGGTTTTGTCTCC | 312 |
| *bla*_GES_ | F: CTCAGATCGGTGTTGCGATC  R: TGTATCTCTGAGGTCGCCAG | 416 |
| *bla*_IMP_ | F: GAAGGCGTTTATGTTCATAC  R: GTACGTTTCAAGAGTGATGC | 587 |
| **Virulence factors encoding Gene** |  | |
| *Kpn* | F: GTATGACTCGGGGAAGATTA  R: CAGAAGCAGCCACCACACG | 626 |
| *entB* | F: ATTTCCTCAACTTCTGGGGC  R: AGCATCGGTGGCGGTGGTCA | 371 |
| *Ycf* | F: ATCAGCAGTCGGGTCAGC  R: CTTCTCCAGCATTCAGCG | 160 |
| *k2A* | F: CAACCATGGTGGTCGATTAG  R: TGGTAGCCATATCCCTTTGG | 543 |
| *magA* | F: GGTGCTCTTTACATCATTGC  R: GCAATGGCCATTTGCGTTAG | 1282 |
| *Uge* | F: TCTTCACGCCTTCCTTCACT  R: GATCATCCGGTCTCCCTGTA | 534 |
| *iutA* | F: GGCTGGACATCATGGGAACTGG  R: CGTCGGGAACGGGTAGAATCG | 300 |
| *ybtS* | F: GACGGAAACAGCACGGTAAA  R: GAGCATAATAAGGCGAAAGA | 242 |
| *rmpA* | F: CATAAGAGTATTGGTTGACAG  R: CTTGCATGAGCCATCTTTCA | 461 |
| *mrkD* | F: AAGCTATCGCTGTACTTCCGGCA  R:GGCGTTGGCGCTCAGATAGG | 340 |
| *fimH* | F: TGCTGCTGGGCTGGTCGATG  R: GGGAGGGTGACGGTGACATC | 688 |
