# Supplementary material for: Co-existence of antibiotic resistance and virulence factors in carbapenem resistant Klebsiella pneumoniae clinical isolates from Alexandria, Egypt
Source: BMC Microbiol. 2024 Nov 11;24:466. doi: 10.1186/s12866-024-03600-1 (PMC11552214; doi:10.1186/s12866-024-03600-1)

**Antimicrobial susceptibility testing (AST)**

**1- Colistin Broth Disk Elution**


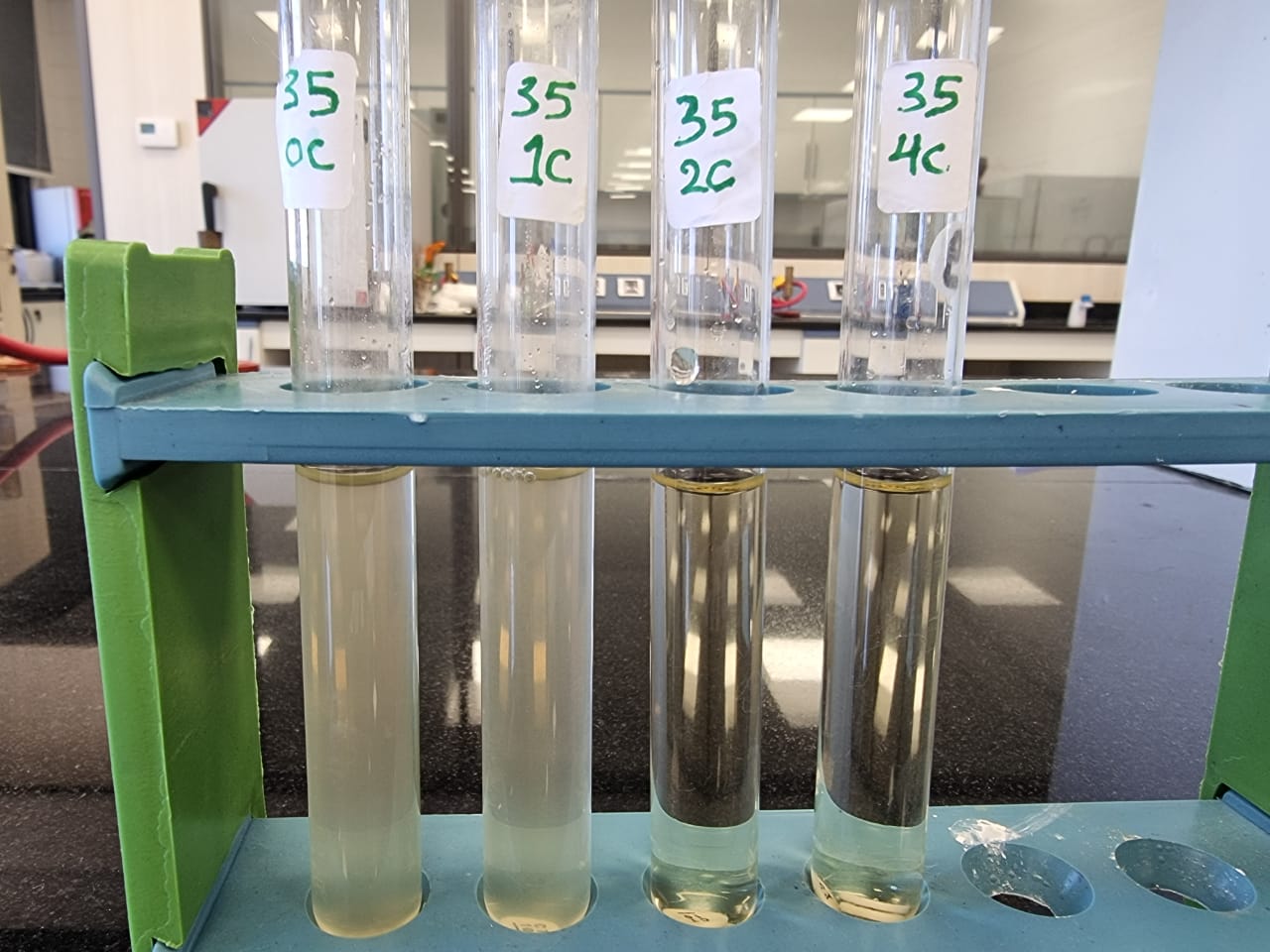


**Fig 1: Sample no 35 shows MIC value = 2 (last clear concentration of colistin)**

**Tubes from left to right** containing 10 mL cation-adjusted Müller-Hinton broth (Oxoid Ltd., Basingstoke, UK) were labelled 0 (as control), 1, 2 and 4 µg/mL. One, two or four colistin (10 µg) discs were transferred to the tubes labeled 1 µg/mL, 2 µg/mL, or 4 µg/mL, respectively. No discs were added to the control tube (0 µg/mL).

**Phenotypic detection of virulence factors**

**1- Biofilm production**


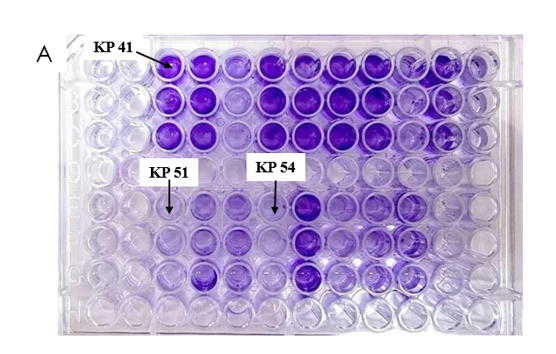


**Fig 2: Biofilm production: Strong biofilm producer (KP41), moderate biofilm producer (KP51) and weak biofilm producer (KP54).**

**2-** **Phospholipase C production (lecithinase production)**


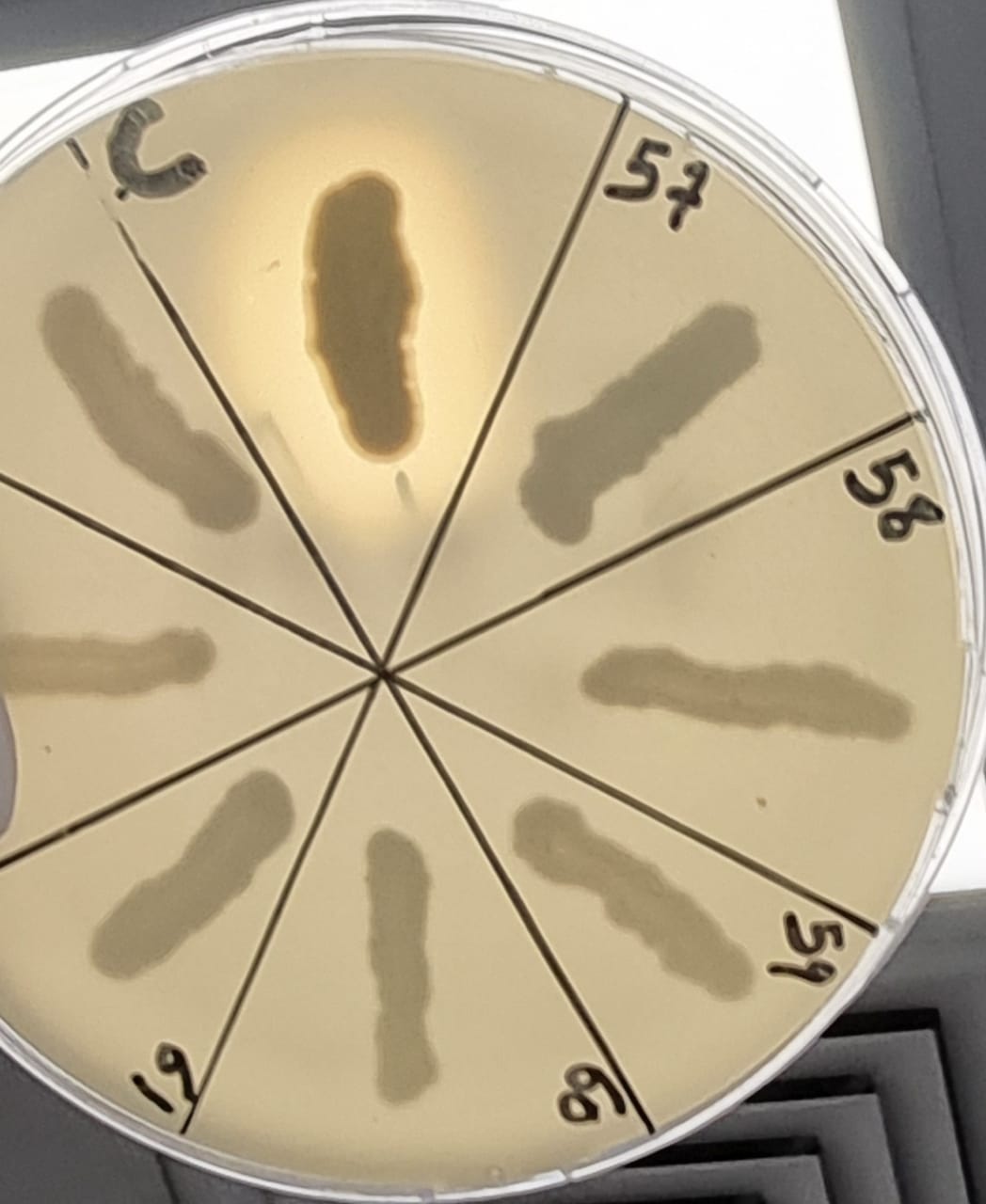


**Phenotypic detection of carbapenemeases**

**1- CarbaNP test**


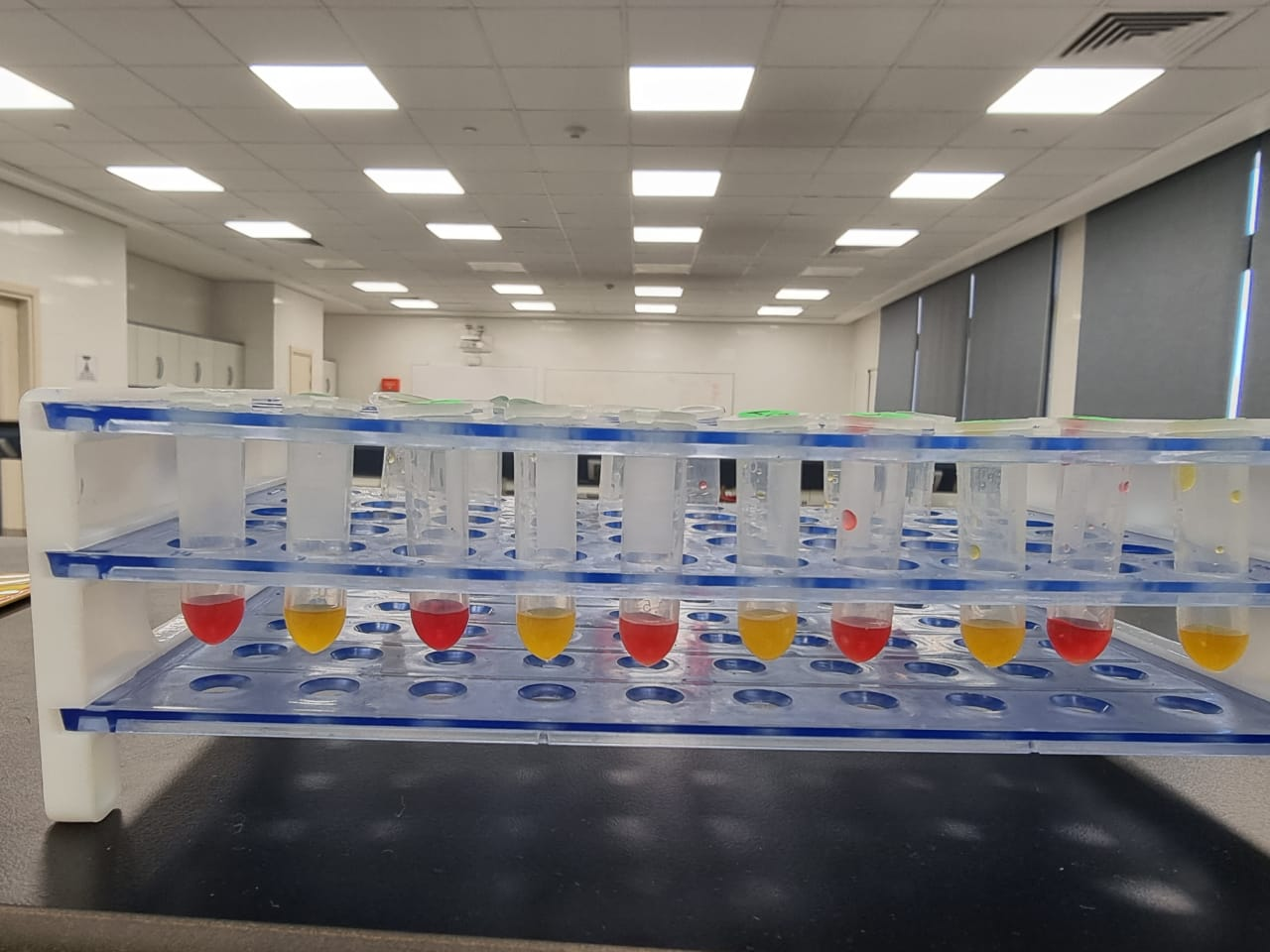


**2- Modified carbapenem inactivation method (mCIM) and EDTA-modified carbapenem inactivation method (eCIM)**


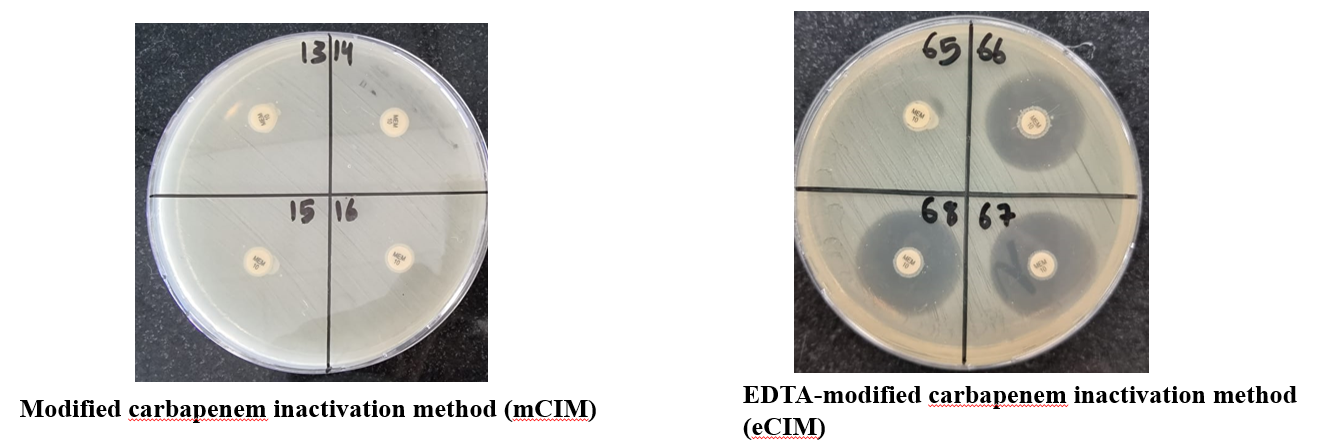


**Genotypic detection of virulence determinants**


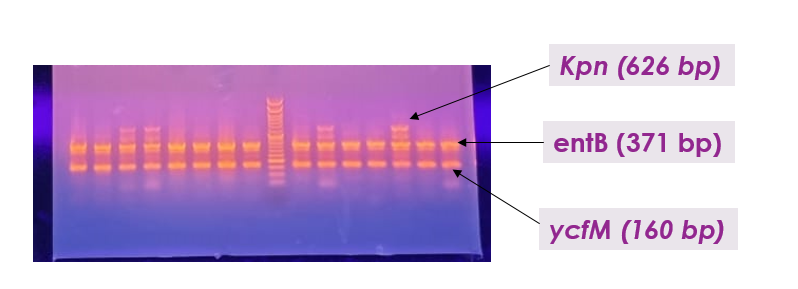


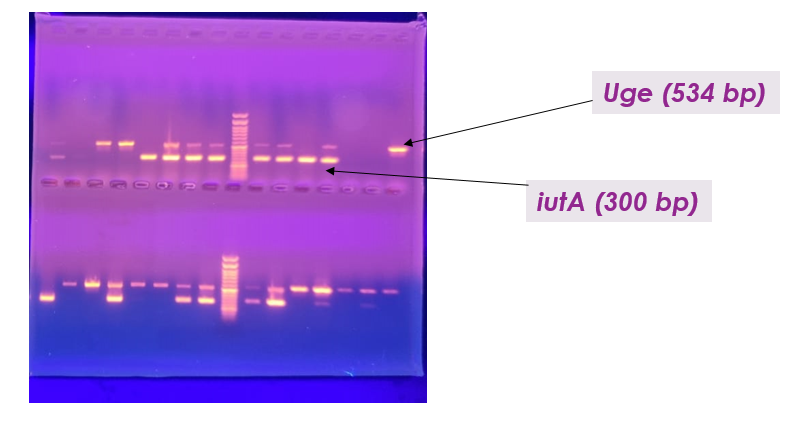


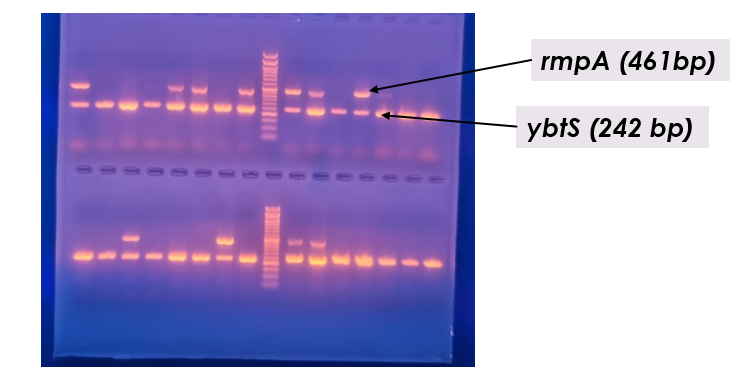


**Genotypic detection of carbapenemeases encoding genes**


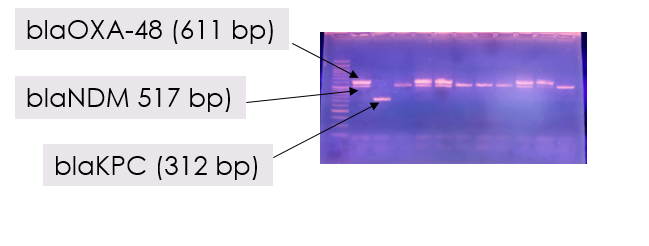

Supplement: Supplementary file 3 — Supplementary Material 3 [file 12866_2024_3600_MOESM3_ESM.docx]
